# Unsupervised Data Mining in nanoscale X-ray Spectro-Microscopic Study of NdFeB Magnet

**DOI:** 10.1038/srep34406

**Published:** 2016-09-29

**Authors:** Xiaoyue Duan, Feifei Yang, Erin Antono, Wenge Yang, Piero Pianetta, Stefano Ermon, Apurva Mehta, Yijin Liu

**Affiliations:** 1 School of computer, Wuhan University, Wuhan, Hubei 430072, China; 2 Division of Biomaterials and Bioengineering, Department of Preventive and Restorative Dental Sciences, UCSF, San Francisco, CA 94143-0758, USA; 3 Department of Computer Science, Stanford University, Stanford, CA 94305-2205, USA; 4 Center for High Pressure Science and Technology Advanced Research, Shanghai 201203, China; 5 Stanford Synchrotron Radiation Lightsource, SLAC National Accelerator Laboratory, Menlo Park, CA 94025, USA

## Abstract

Novel developments in X-ray based spectro-microscopic characterization techniques have increased the rate of acquisition of spatially resolved spectroscopic data by several orders of magnitude over what was possible a few years ago. This accelerated data acquisition, with high spatial resolution at nanoscale and sensitivity to subtle differences in chemistry and atomic structure, provides a unique opportunity to investigate hierarchically complex and structurally heterogeneous systems found in functional devices and materials systems. However, handling and analyzing the large volume data generated poses significant challenges. Here we apply an unsupervised data-mining algorithm known as DBSCAN to study a rare-earth element based permanent magnet material, Nd_2_Fe_14_B. We are able to reduce a large spectro-microscopic dataset of over 300,000 spectra to 3, preserving much of the underlying information. Scientists can easily and quickly analyze in detail three characteristic spectra. Our approach can rapidly provide a concise representation of a large and complex dataset to materials scientists and chemists. For example, it shows that the surface of common Nd_2_Fe_14_B magnet is chemically and structurally very different from the bulk, suggesting a possible surface alteration effect possibly due to the corrosion, which could affect the material’s overall properties.

The ability to investigate material systems that are hierarchically complex and heterogeneously structured offers the possibility of unraveling the interplay of fine-length-scale factors, which are the origins of the material’s macroscopic functionality. While it is often desirable to spatially resolve the fine features within the complex systems, it is usually scientifically even more important to resolve those features with chemical and/or elemental sensitivity. There are quite a number of fine-length-scale probes available for doing this, and they use electrons^1^, X-rays^2^,^3^,^4^,^5^,^6^, visible phonons^7^,^8^, and fine tips^9^ to stimulate and detect the localized signal from the sample. Among these probes, X-ray based methods show advantages in nondestructive study of the sample’s internal structure with the possibility of resolving the chemical species thanks to the X-ray’s penetration capability and chemical sensitivity^10^.

When combined with spectroscopic analysis, the X-ray based full-field imaging^4^ has been recognized as a powerful tool for probing hierarchically complex materials and hence been used in many different fields of research including the studies in energy material^11^,^12^,^13^,^14^,^15^,^16^, industrial catalysis^17^,^18^, and archaeological science^19^. In contrast to bulk X-ray spectroscopic experiments^20^, the use of an area detector in the full-field spectroscopy^2^,^3^,^4^,^5^ allows millions of pixel to work simultaneously. As a result, a large number of spatially resolved spectrums can be acquired in a short time, usually a few million spectra in less than an hour^2^,^4^,^21^. To truly benefit from this advancement in spectro-microscopy, it is essential that some scientifically relevant information is extracted in a comparable time frame. We would like to point out that, in this study, we focus on the full-field transmission X-ray absorption spectroscopic (XAS) imaging method, which is one kind of the technique termed spectro-microscopy that combines the spectroscopic analysis and the spatial resolving capability. However, the above described challenge is applicable to the spectro-microscopy in general because of the similarity in the data structure.

In most cases currently, big datasets from full-field spectro-microscopy are analyzed with heavy dependence on prior knowledge or expectation of known phases. For example, in a study of pressure induced phase transition in a BiNiO_3_ particle^22^, a material that shows negative thermal expansion over a large temperature window^23^, the phase diagram and the signature X-ray absorption spectrum of the end compounds (the pure high pressure phase and the pure low pressure phase) were known *a priori* from bulk X-ray diffraction and spectroscopic measurements. Consequently, it was sufficient to employ the direct linear combination fitting (LCF), a supervised machine learning method, to reconstruct the spatial distribution of these two coexisting phases in the studied particle; and subsequently, to reveal the dynamics of the propagating pressure-driven phase-transition front^22^. This approach is straightforward and fast, yet very powerful, when the end components of the complex systems are known *a priori*. However, often in material science, especially in hierarchically complex system undergoing transformation, many of the end members are not known. The strategy of discovering these unknown phases through a low resolution (or bulk) measurement as was employed for the BiNiO_3_ study is not always successful, because often functionally important phases occur as trace/minority phases, for example, as transition or pinning phases at the reaction front.

Without any guidance from the knowledge of all the end members present in the sample, the analysis of the spectro-microscopic data based on the traditional supervised approach becomes difficult, and tends to overlook functionally important features in complex material systems. Due to the high data acquisition rate, it is not practical to manually interact with every single spectrum as it is acquired. On the other hand, it is risky to simply select a subset of the big data for detailed analysis because the unknown/new material phases are often minority phases spatially segregated in a few small localized region. Automatic clustering algorithms become attractive if they can quickly and reliably find minority phases in spectro-microscopic data. Although clustering algorithms such as k-means clustering^24^ have been applied for unsupervised classification, for example, to the study of LiFePO_4_ battery electrode material^25^ and ancient ceramic samples^26^, they still needs some guidance and inputs from humans, especially about the number of clusters, limiting their applicability. In here, we demonstrate application of the clustering algorithm known as the density-based spatial clustering of applications with noise (DBSCAN)^27^, that is robust to noise and doesn’t require *a priori* knowledge about number of phases.

We apply DBSCAN to analyze full field XAS investigation of a rare-earth element based permanent magnet material, Nd_2_Fe_14_B, which has become the most widely used and one of the strongest types of permanent magnet since it was discovered in 1982^28^. The importance of this type of magnet can be seen from its wide range of industrial applications, e.g. in MRI scanners^29^, Maglev trains^30^, and electric vehicles^31^. Material scientists in this field have been making continuous effort to improve the performance in many aspects including the magnetism, thermal stability, coercivity, etc. Another noticeable effort in this field is the attempt to reduce the use of rare-earth elements due to the concern regarding the stable and sustainable supply of rare-earth elements for the increasing demand^32^. While considerable attention has been paid to the possibility of substituting the rare-earth elements with other materials^33^, the nano/meso scale structural heterogeneity is one of the areas that are believed to have an important impact on the overall performance of the material and, however, are not very well understood. Our analysis efficiently reveals nanoscale heterogeneity in the surface and near surface regions which could affect it’s the durability and macroscopic performance of magnets.

## Results and Discussion

### The NdFeB permanent magnet

**Figure 1:**
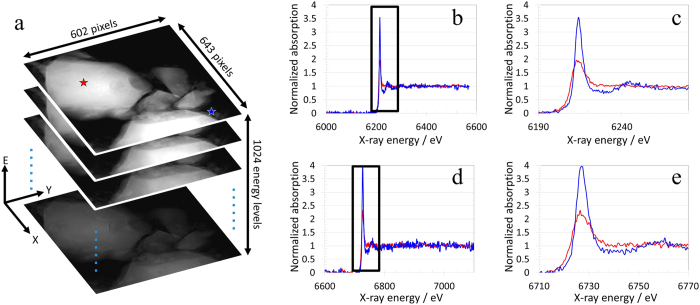
The structure of the nanoscale spectro-microscopic data on the studied Nd_2_Fe_14_B Particle is shown in panel a, indicating it is a three dimensional dataset. The single pixel spectrums over the two highlighted pixels in panel a are shown in panels b (for the Nd L_3_ edge) and d (for the Nd L_2_ edge) with the selected near edge region displayed in panels c and e, respectively. The pixel size of the images is at 31.2 nanometers.

We investigated a small piece of Nd_2_Fe_14_B sample crushed from a magnet rod (Goodfellow, item #531-114-16) using the energy resolved transmission X-ray microscope at beamline 6-2C of the Stanford Synchrotron Radiation Lightsource (SSRL) at SLAC National Accelerator Laboratory. The sample was exposed to the air at room temperature for about a year without any particular effort to protect it from the oxygen and/or humidity, which could cause corrosion on the magnet. Full-field nanoscale spectroscopic imaging was performed over the Nd L_2_ at ~6722 eV and L_3_ edges at ~6208 eV, respectively. We choose to avoid the Nd L_1_ edge at ~7126 eV because it has a much lower cross-section and it largely overlaps with the Fe k edge at ~7112 eV^34^. More details about the experiment can be found in the method section of this work. As shown in Fig. 1, the spectro-microscopic data is set of 2D spatially images collected at 1024 energies over Nd L_3_ and L_2_ edges (or in terms of spectroscopy spatially resolved and energy dependent absorption coefficients). Because of frame-to-frame misalignment images were cropped to 602 × 643 pixels to keep spatially registered over the entire energy stack. The data, therefore, contains over 300,000 points (pixels); and each one of these points has 1026 attributes (2 spatial + 1024 energies). The dataset is not particularly large, but is rather complex due to the very large number of attributes. An Eigen analysis can show that the 1024 energy attributes can be remapped into a few attributes without loss of significant information; still the datasets has over 5 attributes. In conjunction with noise, the complexity of this data poses a challenge.

All the single pixel spectrums over both the Nd L_2_ and L_3_ edges are independently normalized using a well-established method known as MBACK^35^ prior to any further analysis. The normalized spectrums over two selected representative pixels (the red and the blue pixel highlighted in Fig. 1a) are presented in Fig. 1b,d, with the magnified near edge regions shown in Fig. 1c,e, respectively. The near edge region of the spectra shows that the blue pixel near the particle surface is more oxidized than the red one, which is more towards the center of the particle. The differences observed in these two pixel spectrums highlights the nanoscale heterogeneity exist at single pixel (~30 nanometers) level. It worth mentioning that this dataset was acquired within about two hours of beamtime at the beamline 6-2C of SSRL. Given the data acquisition rate at this scale, it is not practical to perform pixel by pixel analysis illustrated in Fig. 1. A robust algorithm is, therefore, very much desired in this study to automatically classify similar spectra (and therefore similar chemical composition) in to a few clusters for further detailed spectroscopic analysis.

### The density-based spatial clustering of applications with noise (DBSCAN)

In order to effectively and automatically perform identification and clustering of the pixels with similar spectrum and, thus, with similar chemical compositions, we adapt a powerful algorithm known as the density-based spatial clustering of applications with noise (DBSCAN)^27^. The spectro-microscopic dataset described above can be viewed as 602 × 632 data points distributed in a high-dimensional space (each attribute of the data can be viewed as an independent dimension). The DBSCAN algorithm evaluates the data density at each location and groups together data points that are densely packed in an area. There are two critical parameters for the algorithm, Eps and MinPts. The Eps parameter represents the radius of the search for neighboring points; the MinPts parameter is the minimum number of data points needed to form a cluster. We briefly describe the idea behind DBSCAN below; see the work by Ester *et al*.^27^ for more details.

**Figure 2:**
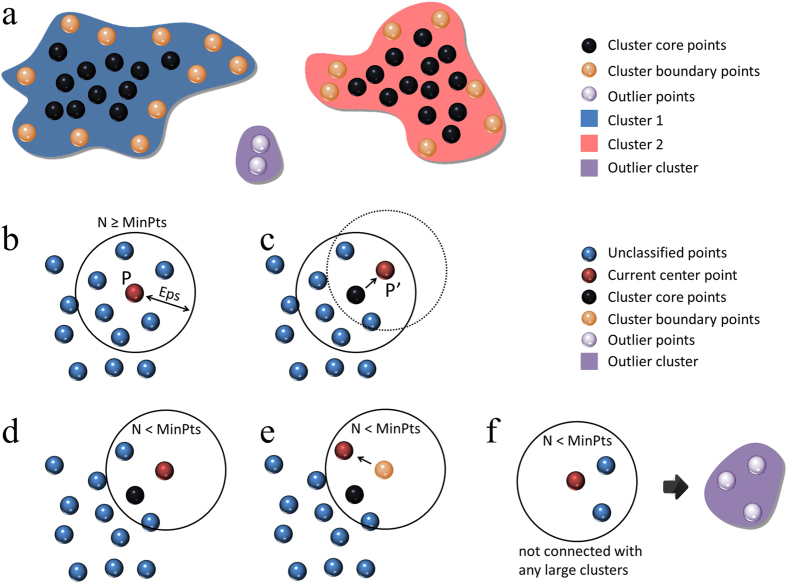
Schematic drawings of the DBSCAN clustering algorithm. Panel a shows the clustering results where the data points are grouped into two major clusters and an outlier group. Panels b through f schematically show how the algorithm searches through the data points distributed in the high-dimensional space.

As shown in Fig. 2a, the data points are grouped into different clusters, with each data point identified as either 1) cluster core point, 2) cluster boundary point, or 3) outlier point. Figure 2b shows a certain iteration of the search centered on data point P. In this step a spherical region in the multi-dimensional space is drawn based on the radius specified by the input parameter Eps. The number of data points within this ball is then calculated. If there are sufficient number of data points (equal to or larger than the input parameter MinPts) within the region, all the points are labelled as belonging to cluster N; the current center point (P) is labelled as a core point of cluster N. After this step, the search center is moved from the current position (P) to another data point (P’) within cluster N, as schematically shown in Fig. 2c. When P’ can be included in cluster N, but cannot be identified as a cluster core point due to the fact that there is an insufficient number of data points within the ball centered at P’ (like the situation shown in Fig. 2d), P’ is identified as a cluster boundary point of cluster N. Figure 2f shows another situation, where there are some data points that cannot be identified as an independent cluster and are not connected with any other large cluster. All those points are regarded as outlier data points. All the outlier data points are grouped together forming an outlier cluster. It worth pointing out that the data points with insufficient signal to noise ratio have been already eliminated from the clustering search. Therefore, outlier data points are not outliers in a traditional sense, but should be regarded as a minority chemical phase in the material. We would mention that the DBSCAN implemented in this work can perform the clustering evaluation rather efficiently. The calculation present in this work takes less than five minutes on a normal laptop.

### The chemical phase maps

**Figure 3:**
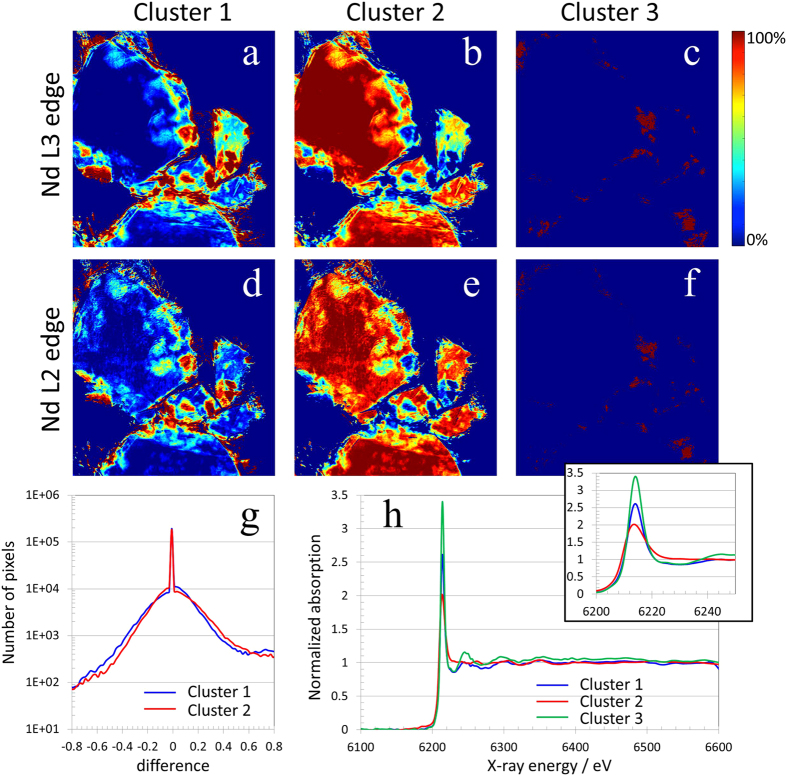
Cluster maps generated by DBSCAN based on the Nd L_3_ edge data are shown in panels a–c, respectively. The cluster maps based on the Nd L_2_ edge data are shown in panels d–f, respectively. Panel g shows the histogram of the difference between the cluster maps in panels a and d (in blue), as well as the difference between panels b and e (in red). A large peak around zero indicates good agreement between the maps generated from these two independent datasets. Panel h shows the averaged spectra for the three identified clusters with the near edge region magnified in the corresponding inset.

As described above, the DBSCAN method is applied to the classification of the spectro-microscopic data of the Nd_2_Fe_14_B sample. For better confidence of the clustering results, we treat L_3_ and L_2_ edges independently. It worth pointing out that L_3_ and L_2_ edges arise from similar electronic excitations and should have very similar spectroscopic signatures^36^. The idea here is to use the uniqueness of the information contained in each edge to crosscheck the end results. In both case, the DBSCAN identified two major chemical components in the sample (phase 1 and phase 2), and a third minority phase (phase 3) which cannot be well represented by either one of these two major phases or their linear combination. The chemical phase maps identified by DBSCAN from the Nd L_3_ edge data are shown in Fig. 3a–c; and the ones from the Nd L_2_ edge data are shown in Fig. 3d–f. The maps in Fig. 3a–c and Fig. 3d–f are similar but not identical. For a more quantitative measurement of the comparison, we show, in Fig. 3g, the histogram of the differences between Fig. 3a,d (in blue), as well as that of Fig. 3b,e (in red). Figure 3g, shows a large peak around zero difference indicating very good agreement between the phase maps generated from the Nd L_3_ edge data and that from the Nd L_2_ edge data (Note, that the histograms are plotted on a log scale in Fig. 3g). Considering the fact that Nd L_3_ edge is significantly stronger than the Nd L_2_ edge, the small number of pixels contributing to the tails of the difference histogram could be attributed to the reduced signal to noise ratio at Nd L_2_ edge. As a result, we will limit our further spectroscopic analysis to the spectra of the three identified chemical phases over the Nd L_3_ edge, which are plotted in Fig. 3h with the near edge region magnified in the corresponding inset. We would also point out that other clustering algorithms, such as the well-known principle component analysis (PCA), can also be used to group the pixels with similar spectroscopic signatures. For comparison we show, in the Supplementary Fig. S1, the PCA generated clustering results of this dataset. In our implementation of PCA, we preserved 98% accuracy of the original data and extracted two principle components. By linear combination fitting of the raw data using the two detected principle components, a third compound, which cannot be well represented by those two major components and their linear combinations, was identified. We again repeated this calculation on both the L_2_ edge and L_3_ edge data. Significant differences in the chemical maps were observed (see Supplementary Fig. S1). As discussed above, the consistency between the chemical maps derived from the L_2_ edge and L_3_ edge data is an important indicator of the figure-of-merit in our study because there is known physical similarity between those two edges. As a result, we conclude that the DBSCAN shows superior performance for unsupervised clustering of the spectro-microscopic data in the presented case study.

An x-ray absorption spectrum contains information about both the electronic structure of the absorbing atom as well as the arrangement of atoms around it. In the clustering analysis so far we have focused on the edge and the near edge structure. The differences between the spectra of the three clusters are most prominent in the intensity and the width of the white-line feature. The intensity and width of the white-line features are thought to be very sensitive to small changes in the p-d orbital hybridization as well as subtle changes in the nearest neighbor distance. The intensity of the white-line can also be suppressed by over-absorption and self-absorption effects, which in turn dependent on the thickness and the porosity of the sample. To address the concern about the thickness effects, we extracted Extended X-ray Absorption Fine Structure (EXAFS) oscillation from the region between the L_3_ and L_2_ edge. (EXAFS oscillations are dominated by the local structure.) Below, we show, based on the analysis of the EXAFS oscillations that the three clusters observed in the spectrums in Fig. 3h also show distinct differences in short-range atomic arrangement around the Nd atoms and are not artifact of variation in the sample thickness.

### EXAFS analysis

**Figure 4:**
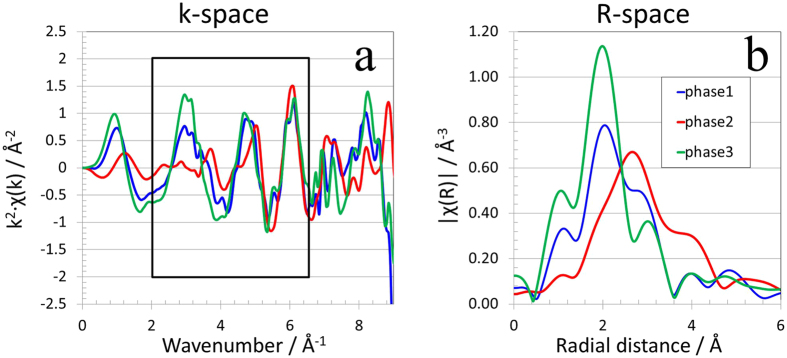
EXAFS analysis of the Nd L_3_ edge spectrums the three chemical phases as identified by the DBSCAN clustering algorithm. Panel a: the k^2^ weighted EXAFS signals in the k-space; panel b: R-space plot calculated using the k-space signal in the wavenumber window of 2 to 6.5.

While the clustering method described above identified three components that appear to have different electronic structure, and provided the spatial distributions of them, the detailed spectroscopic analysis of each one of the three clusters is essential for a deeper understanding of how they differ from each other chemically and structurally. We show in Fig. 4a the EXAFS oscillations of all the three clusters in the momentum space (the k-space) weighted by the factor of k^2^. It is well known that Fourier transformation of the k-space signal can reveal the real space atomic configuration around the targeted central element^37^, which is Nd in this study. We choose to perform Fourier transformation of the k-space data over the selected k range (as indicated by the box in Fig. 4a) to convert it into the real space signal (the R-space, as shown in Fig. 4b) for the purpose of extracting the averaged information of the bonding distance of the first shell coordianted atoms. Figure 4b clearly suggests that the phase 2 has the longest first shell bonding distance, while the phase 3 has the shortest first shell bonding distance. It is interesting to note that the phase 1 shows mixed signature of the phases 2 and 3. The phases 1 and 2 are identified as the major phases because the number of pixels that are assigned to these two clusters are significantly larger than that of phase 3 (as one could see in the Fig. 3a through 3f). However, the EXAFS analysis indicates that the phases 2 and 3 are actually the most chemially different, while phase 1 is likely to be a mixture of these two orthogonal componds. It should be pointed out that the quality of the spectra extracted from our imaging data is not as good as a typical spectrum from bulk spectroscopic measurements due to the limited amount of sample exposed to the beam. We performed bulk EXAFS measurement at beamline 4-1 of SSRL on powdered Nd_2_Fe_14_B sample. As shown in Supplementary Fig. S2, the bulk EXAFS data shows signatures that is similar to that of a mixture phase 1 and 2. Data and analysis of a spectrum from bulk sample, therefore, woundn’t have necessarily suggested that the surface of the sample particles is chemically very different from the core of the particles, nor would it have help identify the spectra needed for a least square reconstruction of the full field x-ray spectra. Our results, thus, highlight not only the the importance of the spatially resolved spectroscopic study but the need for a robust automatic clustering capability, such as that provided by DBSCAN method.

## Summary and Conclusion

The novel developments in the X-ray spectro-microscopic technique have been recognized as a powerful experimental method that has the potential to help solving challenges in many research fields, including investigation of hierarchically complex functional material systems. In order to effectively perform data analysis on modern high-dimensional nanoscale spectro-microscopic experimental datasets, we have introduced an advanced algorithm known as the density-based spatial clustering of applications with noise (DBSCAN). This algorithm can cluster large, high-dimensional datasets with little or no human supervision, automatically discovering important patterns and trends in the data. This method was applied to the study of a rare-earth element based permanent magnet, Nd_2_Fe_14_B. The DBSCAN identified three chemically different compounds as well as their spatial distributions in the studied sample. It is very probable that smaller and more heterogeneously distributed of these phases (phase 3) would have been missed in a traditional supervised analysis. Further detailed spectroscopic investigation suggested that the phases 2 and 3 are the most chemically different, while the phase 1 is likely a mixture of the phases 2 and 3. If phase 1 is indeed a mixture of phase 2 and 3, it shows up as a distinct cluster instead of separating into the two other clusters because it must be mixed at a length scale that is beyond the spatial resolution limit of the current measurement, which is at about 31.2 nm. This finding echoes with the observation of the segregation of a Nd rich phase at the particle surface by Kao *et al*.^34^. It is well known that the sintered Nd_2_Fe_14_B tends to be vulnerable to corrosion, especially along grain boundaries. The particle studied is crashed from a magnet rode, and is likely to be broken away along the existing grain boundaries within the sintered magnet. The surface alternation we observed may also be attributed to the grain boundary corrosion, which can cause serious deterioration. Since the observed Nd rich surface phase increases the consumption of the rare-earth element (Nd) and is altered from the desired composition, it is beneficial to minimize the formation of this undesired secondary phase.

We would also point out that the developed method can also been directly applied to the studies in many other fields, in which the rational design of hierarchically complex functional materials plays an important role, such as catalysts, batteries, fuels cells, and optical devices. Especially with the large investment over last several years in larger and faster detectors, orders of magnitude increase in beam brightness, novel new X-ray optics^38^,^39^,^40^,^41^ and advance X-ray facilities^42^ rate of acquisition of the scientific data has dramatically accelerated. While these new facilities and accelerating rate of new data brings the promise of a deeper understanding of functioning of complex materials under real world conditions, without the ability to perform effective, reliable, and automatic data mining in comparable time frame as data acquisition that promise will remain unfulfilled.

## Methods

We studied a small piece of Nd_2_Fe_14_B sample crushed from a magnet rod (Goodfellow, item #531-114-16) using the energy resolved transmission X-ray microscope (TXM) at beamline 6-2C of the Stanford Synchrotron Radiation Lightsource (SSRL) at SLAC National Accelerator Laboratory.

The TXM experimental setup is briefly described below and more details can be found elsewhere^43^. The X-rays from a 56 pole 0.9 Tesla wiggler pass through several mirrors and are then focused to about 200 microns for serving as the secondary source for the microscope. The monochromator is a liquid nitrogen cooled double-Si(111) crystal system that selects a narrow band pass from the incident X-rays, providing quasi-monochromatic (ΔE/E = 5 × 10^−4^) illumination. The TXM, designed to work over an energy range of ~5–13 keV, utilizes a capillary condenser to focus the beam to a spot with size down to a few of microns for illuminating the sample. A Fresnel zone plate with 200-μm diameter and 30-nm outermost zone width is employed to project to the image of the sample to the detector with magnification factor of around 50. The scintillator crystal after the zone plate converts the incoming X-rays into visible photons and is coupled with an optical lens and detector system further downstream. The spatial resolution of this system is ~30 nm which has been demonstrated in earlier publications^44^.

The projection images of the sample are acquired as the energy of the incoming X-rays is tuned from 6000 eV to 7023 eV with step size at 1 eV, covering the Nd L_2_ at ~6722 eV and L_3_ edges at ~6208 eV. At each energy, one image of the sample as well as one reference image (with the sample moved out of the field of view) are recorded for flat field correction by applying Beer-Lambert law^45^. After the reference correction, the data went through several image processing steps including the magnification correction, the spatial registration of images taken at different energies, and the normalization of the pixel spectrums. All of these were performed using an in-house developed software package known as the TXM-Wizard^21^.

For better reducing the demand of large scale computing power and improving the quality of the clustering calculation, we first reject the pixels with insufficient signal to noise ratio in the corresponding spectrum before loading the entire dataset into the DBSCAN. The limited signal to noise ratio in the rejected pixels is due to the limited amount of sample of interest over the corresponding areas in the field of view. This step allows us to group the outlier pixels (as identified in the DBSCAN) as a third cluster (the outlier cluster, which is also referred to as cluster 3) with confidence, because these pixels provides good signal over the Nd L_3_ and L_2_ edges and thus are, indeed, chemically different from the rest of the pixels.

After clustering the extracted three key spectrums are subjected to spectroscopic analysis using a software package known as Athena^46^ for more detailed investigation.

## Additional Information

**How to cite this article**: Duan, X. *et al*. Unsupervised Data Mining in nanoscale X-ray Spectro-Microscopic Study of NdFeB Magnet. *Sci. Rep.* **6**, 34406; doi: 10.1038/srep34406 (2016).

## Supplementary Material

Supplementary Information

## References

[b1] Goldstein J. I. . Scanning Electron Microscopy and X-ray Microanalysis (Springer: US,, 2003), doi: 10.1007/978-1-4615-0215-9.

[b2] Liu Y. . Applications of Hard X‐ray Full‐Field Transmission X‐ray Microscopy at SSRL. in *10TH Int. Conf. X‐RAY Microsc.* **1365,** 357–360 (AIP Publishing, 2011).

[b3] Guttmann P. . Nanoscale spectroscopy with polarized X-rays by NEXAFS-TXM. Nat. Photonics 6, 25–29 (2011).

[b4] Meirer F. . Three-dimensional imaging of chemical phase transformations at the nanoscale with full-field transmission X-ray microscopy. J. Synchrotron Radiat. 18, 773–781 (2011). 21862859 10.1107/S0909049511019364 PMC3161818

[b5] Rau C., Somogyi A. & Simionovici A. Microimaging and tomography with chemical speciation. Nucl. Instruments Methods Phys. Res. Sect. B Beam Interact. with Mater. Atoms 200, 444–450 (2003).

[b6] Nelson G. J. . Three-dimensional mapping of nickel oxidation states using full field x-ray absorption near edge structure nanotomography. Appl. Phys. Lett. 98, 173109 (2011).

[b7] Rust M. J., Bates M. & Zhuang X. Sub-diffraction-limit imaging by stochastic optical reconstruction microscopy (STORM). Nat. Methods 3, 793–795 (2006). 16896339 10.1038/nmeth929 PMC2700296

[b8] Betzig E. . Imaging intracellular fluorescent proteins at nanometer resolution. Science 313, 1642–1645 (2006). 16902090 10.1126/science.1127344

[b9] Jalili N. & Laxminarayana K. A review of atomic force microscopy imaging systems: application to molecular metrology and biological sciences. Mechatronics 14, 907–945 (2004).

[b10] Sakdinawat A. & Attwood D. Nanoscale X-ray imaging. Nat. Photonics 4, 840–848 (2010).

[b11] Yu Y.-S. . Nonequilibrium Pathways during Electrochemical Phase Transformations in Single Crystals Revealed by Dynamic Chemical Imaging at Nanoscale Resolution. Adv. Energy Mater. 5, 1402040 (2015).

[b12] Gent W. E. . Persistent State-of-Charge Heterogeneity in Relaxed, Partially Charged Li_1− *x*_ Ni_1/3_ Co_1/3_ Mn_1/3_ O_2_ Secondary Particles. Adv. Mater. 28, 6631–6638 (2016). 27187238 10.1002/adma.201601273

[b13] Wang J., Karen Chen-Wiegart Y., Eng C., Shen Q. & Wang J. Visualization of anisotropic-isotropic phase transformation dynamics in battery electrode particles. Nat. Commun. 7, 12372 (2016). 27516044 10.1038/ncomms12372 PMC4990630

[b14] Lin F. . Metal segregation in hierarchically structured cathode materials for high-energy lithium batteries. Nat. Energy 1, 15004 (2016).

[b15] Yang F. . Nanoscale morphological and chemical changes of high voltage lithium-manganese rich NMC composite cathodes with cycling. Nano Lett. 14, 4334–4341 (2014). 25054780 10.1021/nl502090z PMC4134180

[b16] Xu Y. . Structural Integrity – Searching the Key Factor to Supress the Voltage Fade of Li-rich Layered Cathode Materials through 3D X-ray Imaging and Spectroscopy Techniques. Nano Energy, doi: 10.1016/j.nanoen.2016.08.039 (2016).

[b17] Gonzalez-Jimenez I. D. . Hard X-ray Nanotomography of Catalytic Solids at Work. Angew. Chemie 124, 12152–12156 (2012). 10.1002/anie.201204930 23090844

[b18] Cats K. H. . X-ray nanoscopy of cobalt Fischer–Tropsch catalysts at work. Chem. Commun. 49, 4622 (2013). 10.1039/c3cc00160a 23586073

[b19] Meirer F. . Full-field XANES analysis of Roman ceramics to estimate firing conditions—A novel probe to study hierarchical heterogeneous materials. J. Anal. At. Spectrom. 28, 1870 (2013).

[b20] Sharpe L. R., Heineman W. R. & Elder R. C. EXAFS spectroelectrochemistry. Chem. Rev. 90, 705–722 (1990).

[b21] Liu Y. . TXM-Wizard: a program for advanced data collection and evaluation in full-field transmission X-ray microscopy. J. Synchrotron Radiat. 19, 281–287 (2012). 22338691 10.1107/S0909049511049144 PMC3284347

[b22] Liu Y., Wang J., Azuma M., Mao W. L. & Yang W. Five-dimensional visualization of phase transition in BiNiO3 under high pressure. Appl. Phys. Lett. 104, 043108 (2014). 24753622 10.1063/1.4863229 PMC3977758

[b23] Azuma M. . Colossal negative thermal expansion in BiNiO3 induced by intermetallic charge transfer. Nat. Commun. 2, 347 (2011). 21673668 10.1038/ncomms1361 PMC3156814

[b24] MacQueen J. Some methods for classification and analysis of multivariate observations. In Proc. Fifth Berkeley Symp. Math. Stat. Probab. Vol. 1 *Stat.* (The Regents of the University of California, 1967) at http://projecteuclid.org/euclid.bsmsp/1200512992.

[b25] Boesenberg U. . Mesoscale phase distribution in single particles of LiFePO4 following lithium deintercalation. Chem. Mater. 25, 1664–1672 (2013). 23745016 10.1021/cm400106k PMC3670807

[b26] Lerotic M., Mak R., Wirick S., Meirer F. & Jacobsen C. MANTiS: a program for the analysis of X-ray spectromicroscopy data. J. Synchrotron Radiat. 21, 1206–1212 (2014). 25178014 10.1107/S1600577514013964

[b27] Ester Martin, Kriegel Hans-Peter & Jörg Sander X. X. A density-based algorithm for discovering clusters in large spatial databases with noise. *Publ. Proc. 2nd Int. Conf. Knowl. Discov. Data Min.* at http://citeseer.ist.psu.edu/viewdoc/summary? doi=10.1.1.121.9220.

[b28] Herbst J. F., Croat J. J., Pinkerton F. E. & Yelon W. B. Relationships between crystal structure and magnetic properties in Nd 2 Fe 14 B. Phys. Rev. B 29, 4176–4178 (1984).

[b29] Jiang X., Shen G., Lai Y. & Tian J. Development of an Open 0.3 T NdFeB MRI Magnet. IEEE Trans. Appiled Supercond. 14, 1621–1623 (2004).

[b30] Thompson M. T. Practical Issues in the Use of NdFeB Permanent Magnets in Maglev, Motors, Bearings, and Eddy Current Brakes. Proc. IEEE 97, 1758–1767 (2009).

[b31] Wang A., Li H. & Liu C. T. On the Material and Temperature Impacts of Interior Permanent Magnet Machine for Electric Vehicle Applications. IEEE Trans. Magn. 44, 4329–4332 (2008).

[b32] Kramer M. J., McCallum R. W., Anderson I. A. & Constantinides S. Prospects for Non-Rare Earth Permanent Magnets for Traction Motors and Generators. JOM 64, 752–763 (2012).

[b33] Kusne A. G. . On-the-fly machine-learning for high-throughput experiments: search for rare-earth-free permanent magnets. Sci. Rep. 4, 6367 (2014). 25220062 10.1038/srep06367 PMC4163667

[b34] Kao T. L. . Nanoscale elemental sensitivity study of Nd_2_Fe_14_B using absorption correlation tomography. Microsc. Res. Tech. 76, 1112–1117 (2013). 23922210 10.1002/jemt.22273

[b35] Weng T.-C., Waldo G. S. & Penner-Hahn J. E. A method for normalization of X-ray absorption spectra. J. Synchrotron Radiat. 12, 506–510 (2005). 15968130 10.1107/S0909049504034193

[b36] *EXAFS and Near Edge Structure III*. **2** (Springer Berlin Heidelberg, 1984).

[b37] Sayers D. E., Stern E. A. & Lytle F. W. New Technique for Investigating Noncrystalline Structures: Fourier Analysis of the Extended X-Ray—Absorption Fine Structure. Phys. Rev. Lett. 27, 1204–1207 (1971).

[b38] Nazaretski E. . Development and characterization of monolithic multilayer Laue lens nanofocusing optics. Appl. Phys. Lett. 108, 261102 (2016).

[b39] Yan H., Conley R., Bouet N. & Chu Y. S. Hard x-ray nanofocusing by multilayer Laue lenses. J. Phys. D. Appl. Phys. 47, 263001 (2014).

[b40] Chang C. & Sakdinawat A. Ultra-high aspect ratio high-resolution nanofabrication for hard X-ray diffractive optics. Nat. Commun. 5, 4243 (2014). 24970569 10.1038/ncomms5243

[b41] Huang X. . 11 nm hard X-ray focus from a large-aperture multilayer Laue lens. Sci. Rep. 3, 204801 (2013). 10.1038/srep03562 PMC3868962 24356395

[b42] Eriksson M., van der Veen J. F. & Quitmann C. Diffraction-limited storage rings - a window to the science of tomorrow. J. Synchrotron Radiat. 21, 837–842 (2014). 25177975 10.1107/S1600577514019286

[b43] Liu Y. . Phase retrieval using polychromatic illumination for transmission X-ray microscopy. Opt. Express 19, 540–545 (2011). 21263593 10.1364/OE.19.0540 PMC3482903

[b44] Andrews J. C. . Nanoscale X-ray microscopic imaging of mammalian mineralized tissue. Microsc. Microanal. 16, 327–336 (2010). 20374681 10.1017/S1431927610000231 PMC2873966

[b45] Swinehart D. F. The Beer-Lambert Law. J. Chem. Educ. 39, 333 (1962).

[b46] Ravel B. & Newville M. ATHENA, ARTEMIS, HEPHAESTUS: data analysis for X-ray absorption spectroscopy using IFEFFIT. J. Synchrotron Radiat. 12, 537–541 (2005). 15968136 10.1107/S0909049505012719

