# Supplementary material for: Unsupervised Data Mining in nanoscale X-ray Spectro-Microscopic Study of NdFeB Magnet
Source: Sci Rep. 2016 Sep 29;6:34406. doi: 10.1038/srep34406 (PMC5041149; doi:10.1038/srep34406)
Supplement: Supplementary Information [file srep34406-s1.pdf]

Supplementary Information:

Unsupervised Data Mining in nanoscale X-ray Spectro-Microscopic Study of NdFeB Magnet

Xiaoyue Duan[1], Feifei Yang[2], Erin Antono[3], Wenge Yang[4], Piero Pianetta[5], Stefano Ermon[3], Apurva Mehta[5,*], Yijin Liu[5,*]

[1]School of computer, Wuhan University, Wuhan, Hubei 430072, China.

[2]Division of Biomaterials and Bioengineering, Department of Preventive and Restorative Dental Sciences, UCSF, San Francisco, CA 94143-0758, USA

[3]Department of Computer Science, Stanford University, Stanford, CA 94305-2205, USA.

[4]Center for High Pressure Science and Technology Advanced Research, Shanghai 201203, China.

[5]Stanford Synchrotron Radiation Lightsource, SLAC National Accelerator Laboratory, Menlo Park, CA 94025, USA.

[*]Correspondence and requests for materials should be addressed to Y.L. (liuyijin@slac.stanford.edu) and A.M. (mehta@slac.stanford.edu)

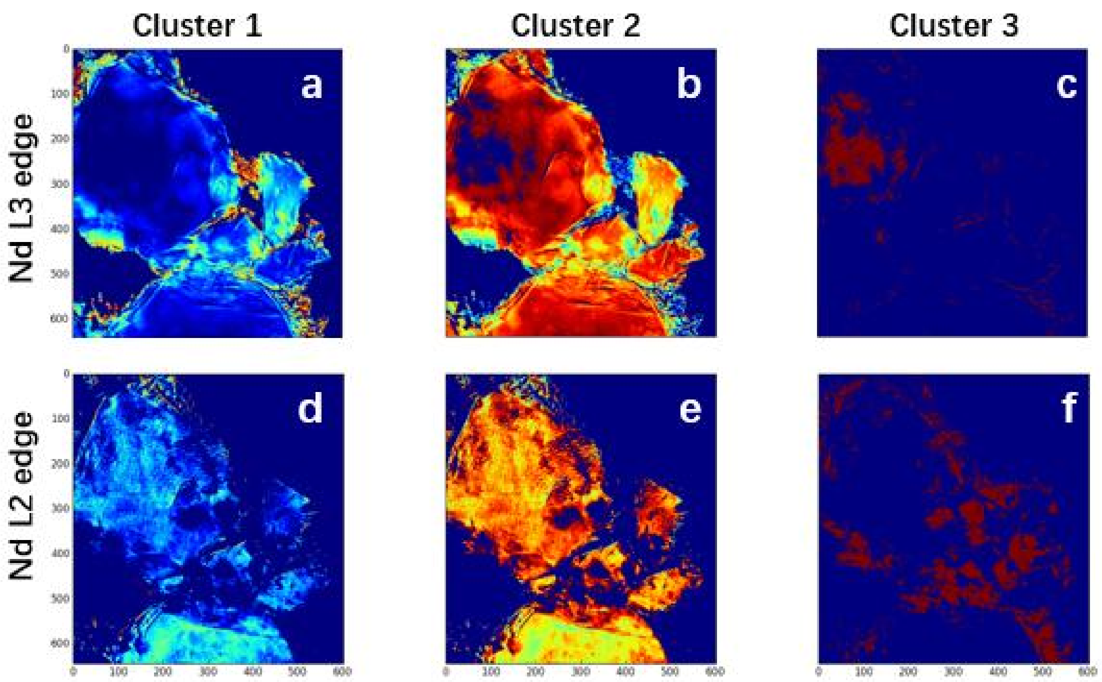

Figure S1. Cluster maps generated by applying PCA. The maps associated with the Nd L3 edge data are shown in panels a, b, and c. The maps associated with the Nd L2 edge data are shown in panels d, e, and f, respectively. Significant differences can be observed between the top row and the bottom row, indicating the PCA clustering failed to satisfy the consistency between the Nd L3 and L2 edges in the presented case study, which is a known physical constrain.

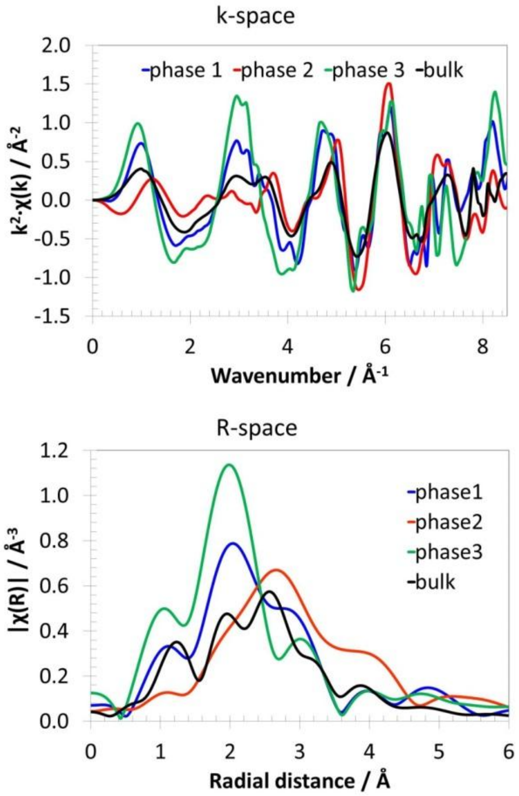

Figure S2. EXAFS analysis of the Nd L3 edge spectrums the three chemical phases as identified by the DBSCAN clustering algorithm and the bulk spectrum. Panel a: the k^2 weighted EXAFS signals in the k-space; panel b: R-space plot calculated.
